# Effect of different levels of green tea (*Camellia sinensis*) and mulberry (*Morus alba*) leaves powder on performance, carcass characteristics, immune response and intestinal morphology of broiler chickens

**DOI:** 10.1002/vms3.1133

**Published:** 2023-04-03

**Authors:** Fatemeh Aziz‐Aliabadi, Hadi Noruzi, Ahmad Hassanabadi

**Affiliations:** ^1^ Faculty of Agriculture Department of Animal Science Ferdowsi University of Mashhad Mashhad Iran

**Keywords:** broilers, green tea, immune response, intestinal morphology, mulberry leaf, performance

## Abstract

**Background:**

In recent years, the use of medicinal plants as an alternative to antibiotics has expanded. Plants containing medicines and antioxidants can improve the performance of poultry.

**Objectives:**

The purpose of this study was to achieve the appropriate levels of green tea leaf powder (GTP) and mulberry leaf powder (MLP) in the diet, which positively affects broilers’ performance.

**Methods:**

648 one‐day‐old Ross 308 broiler chickens were allocated to nine dietary treatments with six replicates and each replicate containing 12 birds based on a completely randomised design (CRD) in a factorial arrangement of 3 × 3 with three levels of GTP, and three levels of MLP for 42 days. Treatments included: (1) no GTP + no MLP (control), (2) 1% GTP + no MLP, (3) 2% GTP + no MLP, (4) no GTP + 1% MLP, (5) 1% GTP + 1% MLP, (6) 2% GTP + 1% MLP, (7) no GTP + 2% MLP, (8) 1% GTP + 2% MLP and (9) 2% GTP + 2% MLP.

**Results:**

The results revealed that the effect of added powders (2%) was significant and increased daily weight gain (DWG) and reduced feed conversion ratio (FCR) compared to the control group during the grower and whole phases (*p* < 0.05). On 35 days, the control and 2% GTP + 2% MLP treatment had the lowest and highest antibodies titre (total and immunoglobulin G [IgG]), respectively (*p* < 0.05). The groups fed with 1% GTP + 1% MLP showed higher villus height (VH) compared to the control, 2% GTP + 1% MLP, 1% GTP + 2% MLP, and 2% GTP + 2% MLP groups (*p* < 0.05). The ratio of the villus height to crypt depth (VH: CD) in treatments 1% GTP + no MLP, 2% GTP + no MLP and 1% GTP + 1% MLP was significantly higher than the control treatment (*p* < 0.05).

**Conclusions:**

It was concluded that the addition of 2% GTP or MLP could improve humoral immune response and performance, and the addition of 1% GTP without MLP increased VH: CD in broilers.

## Graphical abstract

648 one‐day‐old Ross 308 broiler chickens were allocated to nine treatments with three levels of green tea or mulberry leaves powder for 42 days.

The levels of green tea or mulberry leaves powder were 0%, 1% and 2%.

The addition of 2% green tea leaf powder or mulberry leaf powder could improve humoral immunity and performance, and the addition of 1% green tea leaf powder without mulberry leaf powder increased villus height to crypt depth ratio.

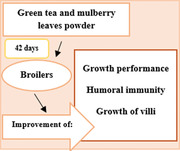

## 1 INTRODUCTION

Nowadays, to increase the performance in the poultry industry as well as the production of chemical‐free meat, the use of natural additives has been considered by broiler farmers. Herbal supplements containing antioxidants can improve growth performance and immune response in chickens (Romeo et al., 2009). In order to improve production performance in livestock, leaves of medicinal plants (powder or extract), spices, and other related products are used as a substitute for antibiotics. These compounds can to activate digestive enzymes and improve performance (Brenes & Roura, 2010).

Tea (*Camellia sinensis L*.) is an evergreen plant with white flowers and green fruits with two to three seeds (Khan, 2014). Green tea is nutritionally valuable, encompasses urgent nutrients, including amino acids, of which L‐theanine accounts for more than half of the total amino acids (Khan, 2014), and has polyphenol catechin (Angga et al., 2018). Green tea is very healthy and non‐toxic nutritious; it has been found that green tea contains large amounts of beneficial antioxidants (Wolfram, 2007). White berry (*Morus alba*) is the name of a species of berry genus; mulberry leaves are rich in protein (15–35%), minerals (calcium: 2.42–4.71%; phosphorus: 0.23–0.97%), energy (1.130–2.240 kcal/kg) and very few (negligible) anti‐nutritional factors (Sarita et al., 2006). Plants rich in flavonoids, such as green tea, mulberry leaves and thyme with, have antibacterial effects that improve the performance and immune system (Cook & Samman, 1999). Although carcass efficiency is significantly affected by age and genetics, carcass composition is also greatly affected by diet (Esmail, 1999). Yang et al. (2003) Compared different levels of green tea by‐products (0.5%, 1% and 2%) with antibiotics and obtained significant results regarding body weight gain. Shomal et al. (2012) stated that performance and health of broilers improved when diets containing green tea were consumed. These advantageous effects can be attributed to the presence of bioactive compounds, such as catechins, flavonoids and flavonols in green tea (Khan, 2014). The attendance of phenolic acids such as tannins in the green tea leaf could intervene with the utilisation of protein and other nutrients, and potentially damaging the performance of animals (Mahlak et al., 2021). Hence, it is momentous that a maximum inclusion level of green tea in broilers’ diets must be identified so as not to compromise their performance. On the other hand, because of the great amount of crude fibre in mulberry leaves and the presence of anti‐nutritional factors such as tannin, the excessive addition of mulberry leaves could affect the performance and health of poultry, which, to a certain extent, restricts it is too much use in animal production (Srivastava et al., 2006). Thus, this study aimed to achieve the appropriate levels of green tea leaf powder (GTP) and mulberry leaf powder (MLP) in the diet, which positively affects broilers’ performance.

## 2 MATERIALS AND METHODS

### 2.1 Preparation of green tea and mulberry leaves powder

Dry green tea and mulberry leaves were purchased from an herbal pharmacy in Rasht‐Iran. Samples were ground using a Grinder (Kinematica AG, Malters, Switzerland) to a particle size of 2 mm. The green tea and mulberry leaf powders were analysed to determine the amount of dry matter (DM), crude protein (CP), crude fibre (CF) and ash (AOAC, 2005). The results of the approximate analysis are shown in Table 1.

**TABLE 1:** Approximate analysis for dried green tea and mulberry leaves powder.

Item	DM (%)	CP (%)	CF (%)	Ash (%)
GTP	91.46	19.34	13.29	5.65
MLP	91.77	16.80	14.95	10.12

DM: dry matter; CP: crude protein; CF: crude fibre; GTP: green tea powder; MLP: mulberry leaf powder.

**TABLE 2:** Ingredients and nutrient composition of the experimental diets (as‐fed basis).

Ingredients (%)	Starter (1−10 days)	Grower (11−24 days)	Finisher (25−42 days)
Corn	50.77	54.12	59.39
Soybean meal (44%)	38.62	34.86	29.41
Fish meal	2.00	2.00	2.00
Soybean oil	4.12	5.00	5.54
Limestone	1.07	0.98	0.90
Dicalcium phosphate	1.88	1.66	1.45
Common salt	0.27	0.27	0.27
Vitamin premix^1^	0.25	0.25	0.25
Mineral premix^2^	0.25	0.25	0.25
L‐Lysine HCl	0.22	0.14	0.14
DL‐Methionine	0.36	0.30	0.26
L‐Threonine	0.10	0.07	0.05
Sodium bicarbonate	0.10	0.10	0.10
Nutrient composition (%)			
Metabolisable energy (kcal/kg)	3000	3100	3200
Crude protein	23	21.5	19.5
Lysine	1.44	1.29	1.15
Methionine + cystine	1.08	0.99	0.90
Threonine	0.97	0.88	0.78
Calcium	0.96	0.87	0.78
Available phosphorus	0.48	0.435	0.39
Sodium	0.16	0.16	0.16
Potassium	0.94	0.87	0.78
Chlorine	0.21	0.21	0.21

^1^ Provided the followings per kg of diet: vitamin A (trans‐retinyl acetate), 12500 U; vitamin D3 (cholecalciferol), 5000 U; vitamin E (D L‐α tocopherol acetate), 80 U; vitamin K (menadione), 3.20 mg; riboflavin, 8.6 mg; pantothenic acid (D‐Ca pantothenate), 18.6 mg; pyridoxine (pyridoxine‐HCl), 4.86 mg; thiamine, 3.2 mg; vitamin B12 (cyanocobalamin), 0.02 mg; biotin, 0.25 mg; folic acid, 2.2 mg; nicotinic acid, 62.51 mg; choline chloride, 500 mg; ethoxyquin (antioxidant), 2.5 mg.

^2^ Provided the following per kg of diet: Fe, 20.23 mg; Zn, 110 mg; Mn, 120 mg; Cu, 16 mg; I, 1.25 mg; Se, 0.30 mg.

### 2.2 Birds, diets and housing

A total of 648 one‐day‐old Ross 308 broiler male chicks with average weights of 36.12 ± 0.97 g were obtained from a local commercial hatchery and reared in pens (1 m × 1 m) for 42 days. Twelve birds were placed in each pen. Nine experimental diets (3 × 3 with six replicates) were fed to the birds during the starter (1–10 days of age), grower (11–24 days of age) and finisher (25–42 days of age) phases. The treatments included (1) no GTP + no MLP (control diet), (2) 1% GTP + no MLP, (3) 2% GTP + no MLP, (4) no GTP + 1% MLP, (5) 1% GTP + 1% MLP, (6) 2% GTP + 1% MLP, (7) no GTP + 2% MLP, (8) 1% GTP + 2% MLP and (9) 2% GTP + 2% MLP.

The nutrient composition of feed ingredients published (NRC, 1994) was utilised for feed formulation. Ingredients and nutrient composition of diets are shown in Table 2. All diets were formulated based on the Ross 308 nutrition guidelines (Aviagen, 2014a). The rearing house temperature was stabilised at 32°C in the first three days and reduced by 3°C every week to reach 21°C and stayed constant until the end of the trial period. Relative humidity was held between 50% and 60% during the trial period. Light and darkness were set at 18 h light and 6 h dark all over the experiment. Feed and water were provided ad libitum all through the experiment. Management practices were done based on the recommendations (Aviagen, 2014b).

### 2.3 Growth performance

The chickens in each pen were weighed collectively at 11, 24 and 42 days of age. The daily weight gain (DWG) and daily feed intake (DFI) were determined for each pen. Feed intake was measured by subtracting the remaining feed from the offered feed in each pen during each study period. Feed conversion ratio (FCR) was corrected for mortality and represented as grams of feed consumed by all chickens in each pen divided by grams of body weight gain. Mortality for each pen was recorded daily.

### 2.4 Carcass characteristics

At 42 days of age, two birds per pen were selected, weighed and decapitated to measure carcass yield. The liver, spleen, bursa of Fabricius and abdominal fat were weighed separately. The results were expressed as a per cent of live weight.

### 2.5 Immune response

#### 2.5.1 Blood sampling

To evaluate the humoral immune response, chickens were immunised intramuscularly (breast muscle) with 0.1 mL of 25% sheep red blood cell (SRBC) in phosphate buffered saline (PBS) on days 8 and 22 posthatching (Dietert, 2009). Blood samples were collected from chickens (two birds in each pen) via the wing vein on days 21, 28, 35 and 42 of age. Blood samples were kept at room temperature for 3 h and then at 4°C overnight. They were centrifuged for 10 min at 2500 × *g*, and sera were separated. Then the sera were frozen and stored at −20°C for later laboratory steps. The anti‐SRBC titres for total antibody and immunoglobulin G (IgG) were determined by using the haemagglutination (HA) method. The serum samples were thawed and placed at room temperature. Then, to inactivate the complement, sera were incubated for 30 min at 56°C. immunoglobulin G titre was determined by incubating the serum with 25 µl of 0.2 Molar 2‐mercaptoethanol (Immunoglobulin M (IgM) is sensitive to 2‐mercaptoethanol and is degraded in its presence) in PBS for 1 h at 37°C. The levels of IgM were obtained from the difference between the IgG and total anti‐SRBC titres. Antibody titres were represented as log_2_ of the reciprocal of the highest dilution giving visible agglutination (Dietert, 2009).

### 2.6 Intestinal morphology

Two birds from each pen were haphazardly selected and euthanised by cervical dislocation at 42 days of age. The entire intestinal tract was pick up, and pieces of approximately 1 cm were taken from the middle part of the jejunum. The pieces were fixed in 10% neutral buffered formalin solution and infixed in paraffin wax later. All histological morphometric studies are accomplished on 5 µm sections and stained with haematoxylin and eosin. To study the morphology of the tissue samples, a computer‐connected optical microscope (Olympus model BX51 microscope; magnification 100) was used to obtain images of the samples, and consequently, the parameters including VH, villus width (VW), crypt depth (CD), and the thickness of muscle layer were measured using the relevant software (Garcia et al., 2007).

### 2.7 Statistical analyses

This trial was performed based on a completely randomised design (CRD) in a factorial arrangement of 3 × 3 with three levels of GTP and three levels of MLP with six replicates by the GLM^1^ procedure of SAS 9.4 (2012) software. Results are showed as means ± standard error of the mean (SEM). All data were normalised by the Shapiro–Wilk test. The significance of treatment differences was analysed using ANOVA. The means were compared by Tukey's test (*p* < 0 .05).

## 3 RESULTS

### 3.1 Growth performance

The results of DWG, DFI and FCR for the starter, grower, and finisher phases are indicated in Table 3. Different levels of GTP and MLP did not significantly affect DFI (*p* > 0.05). However, the main effects of GTP and MLP were significant (*p* < 0.05). Thereby, the groups receiving 2% GTP or MLP showed more DWG compared to the control group (*p* < 0.05). However, no significant difference was observed between the groups receiving 1% and 2% GTP or MLP (during the grower and whole phases) (*p* > 0.05). Regarding the main effects of GTP and MLP on FCR, the treatments receiving 2% GTP or MLP showed a better FCR compared to the control treatment (during the grower and whole phases) (*p* < 0.05). The interactions were not significant (*p* > 0.05).

**TABLE 3:** Effects of different levels of green tea (GTP) and mulberry leaf powder (MLP) on the performance of broilers during the starter, grower and finisher phases.

Treatments	Starter (1–10 days of age)			Grower (11–24 days of age)			Finisher (25–42 days of age)			Whole phase (1–42 days of age)		
Main effects^*^	DWG (g/bird/day)	DFI (g/bird/day)	FCR (g:g)	DWG (g/bird/day)	DFI (g/bird/day)	FCR (g:g)	DWG (g/bird/day)	DFI (g/bird/day)	FCR (g:g)	DWG (g/bird/day)	DFI (g/bird/day)	FCR (g:g)
GTP (%)												
0	19.10	25.34	1.33	45.64^b^	67.94	1.50^a^	79.65	143.16	1.80	53.89^b^	90.03	1.67^a^
1	19.06	25.27	1.33	47.20^ab^	68.38	1.45^ab^	79.24	142.48	1.80	54.22^ab^	89.87	1.65^ab^
2	19.17	25.17	1.31	48.25^a^	68.84	1.41^b^	79.39	143.42	1.79	54.67^a^	90.64	1.63^b^
*p* Value	0.43	0.18	0.17	0.002	0.87	0.02	0.29	0.21	0.77	0.02	0.63	0.02
SEM	0.064	0.061	0.006	0.499	0.762	0.022	0.184	0.326	0.004	0.183	0.286	0.007
MLP (%)												
0	19.08	25.31	1.33	45.99^b^	69.42	1.51^a^	79.29	142.79	1.80	53.85^b^	90.36	1.68^a^
1	19.13	25.17	1.32	47.41^ab^	67.31	1.42^b^	79.35	142.46	1.79	54.36^ab^	89.48	1.66^ab^
2	19.11	25.31	1.32	47.69^a^	69.44	1.41^b^	79.63	142.80	1.79	54.58^a^	91.70	1.64^b^
*p* Value	0.86	0.20	0.38	0.04	0.11	0.006	0.38	0.70	0.42	0.02	0.09	0.03
SEM	0.064	0.061	0.006	0.499	0.762	0.022	0.184	0.326	0.004	0.183	0.286	0.007
Interaction effects												
no GTP + no MLP	18.99	25.48	1.34	44.34	68.67	1.55	78.98	143.11	1.81	53.15	90.29	1.70
1% GTP + no MLP	18.94	25.14	1.33	46.53	70.64	1.52	79.26	142.86	1.80	53.99	90.75	1.68
2% GTP + no MLP	19.31	25.31	1.31	47.11	68.95	1.47	79.63	142.41	1.78	54.43	90.04	1.65
no GTP + 1% MLP	19.15	25.10	1.32	45.73	67.67	1.48	80.03	143.23	1.79	54.10	89.91	1.66
1% GTP + 1% MLP	19.23	25.30	1.32	47.29	66.75	1.41	78.99	141.91	1.80	54.19	89.09	1.64
2% GTP + 1% MLP	19.02	25.12	1.32	49.20	67.51	1.37	79.03	142.23	1.80	54.80	89.44	1.63
no GTP + 2% MLP	19.14	25.44	1.33	46.86	67.50	1.45	79.93	143.13	1.79	54.43	89.90	1.65
1% GTP + 2% MLP	19.00	25.39	1.34	47.76	67.76	1.42	79.46	142.66	1.79	54.50	89.77	1.65
2% GTP + 2% MLP	19.19	25.11	1.31	48.45	67.06	1.38	79.50	142.61	1.79	54.79	89.42	1.63
*p* Value	0.11	0.06	0.23	0.79	0.84	0.96	0.12	0.89	0.23	0.58	0.74	0.89
SEM	0.116	0.105	0.010	0.864	1.319	0.038	0.318	0.564	0.007	0.316	0.495	0.013

^a,b^Means within a column without a common superscript significantly differ (*p* < 0.05).

DWG: daily weight gain; DFI: daily feed intake; FCR: feed conversion ratio; SEM: standard error of mean.

^*^0, 1, 2: different levels of GTP and MLP (%).

### 3.2 Carcass characteristics

The results of carcass characteristics percentage at 42 days of age are presented in Table 4. Carcass characteristics were not affected by experimental treatments (*p* > 0.05).

**TABLE 4:** Effects of different levels of green tea (GTP) and mulberry leaf powder (MLP) on carcass characteristics (%) of broilers at 42 days of age.

Main effects^*^	Carcass	Breast	Thigh	Liver	Spleen	Bursa of Fabricius	Thymus	Abdominal fat
GTP (%)								
0	63.78	23.98	18.16	2.15	0.13	0.15	0.28	1.23
1	63.95	24.42	18.22	1.96	0.12	0.16	0.29	1.28
2	63.44	24.43	18.62	2.03	0.12	0.17	0.30	1.29
*p* Value	0.70	0.68	0.29	0.08	0.39	0.69	0.69	0.31
SEM	0.429	0.412	0.225	0.059	0.006	0.011	0.011	0.085
MLP (%)								
0	63.10	24.66	18.40	2.06	0.13	0.16	0.29	1.41
1	63.57	24.37	18.62	2.05	0.12	0.16	0.28	1.36
2	64.49	23.81	17.99	2.02	0.13	0.15	0.29	1.34
*p* Value	0.08	0.34	0.15	0.89	0.35	0.87	0.89	0.11
SEM	0.429	0.412	0.225	0.059	0.006	0.011	0.011	0.085
Interaction effects								
no GTP + no MLP	63.67	24.07	18.27	2.22	0.13	0.16	0.29	1.41
1% GTP + no MLP	62.79	24.97	17.66	1.95	0.12	0.16	0.29	1.47
%2 GTP + no MLP	62.84	24.94	19.27	2.02	0.11	0.15	0.28	1.49
no GTP + 1% MLP	62.82	23.84	18.23	2.14	0.14	0.14	0.27	1.39
1% GTP + 1% MLP	64.01	23.66	19.00	1.98	0.11	0.16	0.29	1.30
2% GTP + 1% MLP	63.89	25.61	18.62	2.04	0.11	0.17	0.30	1.25
no GTP + 2% MLP	64.84	24.04	17.98	2.09	0.13	0.15	0.28	1.41
1% GTP + 2% MLP	65.05	24.62	18.02	1.95	0.15	0.17	0.30	1.35
2% GTP + 2% MLP	63.61	22.71	17.99	2.02	0.13	0.17	0.30	1.48
*p* Value	0.43	0.10	0.11	0.95	0.31	0.86	0.85	0.23
SEM	0.744	0.713	0.389	0.103	0.011	0.018	0.018	0.147

SEM: standard error of mean.

^*^0, 1, 2: different levels of GTP and MLP (%).

### 3.3 Immune response

The data related to the main and interaction effects of GTP and MLP on the total Anti‐SRBC, IgG, and IgM on days 21, 28, 35 and 42 are shown in Table 5. On 35 days, the main effects of GTP and MLP on total anti‐SRBC and IgG showed a remarkable difference (*p* < 0.05). Thereby, the birds receiving 1% and 2% GTP or MLP showed an increment in total anti‐SRBC titre and IgG. Regarding the interaction effects of GTP and MLP, the total anti‐SRBC and IgG titres in the control treatment were significantly lower than other treatments (*p* < 0.05). The highest total anti‐SRBC titre was observed in treatments: 2% GTP + 2% MLP, 1% GTP + 1% MLP, 1% GTP + no MLP and no GTP + 2% MLP, which was notably superior than the control group (*p* < 0.05). Treatments containing GTP and MLP showed higher IgG titre than the control group (*p* < 0.05). This showed that adding of GTP and MLP increased the IgG titre regardless of the levels used. However, the treatments containing 2% GTP + 2% MLP, 1% GTP + 1% MLP, 1% GTP + no MLP, no GTP + 2% MLP and 2% GTP + no MLP showed the highest IgG titre (*p* < 0.05).

**TABLE 5:** Effects of different levels of green tea (GTP) and mulberry leaf powder (MLP) on total antibody titres, IgG and IgM (log2).

Experimental treatments	Sampling days											
	Total antibody titre				IgM				IgG			
Main effects^*^	21	28	35	42	21	28	35	42	21	28	35	42
GTP (%)												
0	5.14	6.36	6.27^b^	5.46	2.44	2.18	2.80	2.75	2.70	4.18	3.47^b^	2.71
1	5.14	6.37	6.34^a^	5.45	2.43	2.20	2.82	2.75	2.72	4.17	3.53^a^	2.70
2	5.16	6.36	6.36^a^	5.47	2.45	2.18	2.81	2.77	2.71	4.18	3.55^a^	2.70
*p* Value	0.23	0.27	<.0001	0.24	0.28	0.11	0.07	0.08	0.09	0.23	0.03	0.26
SEM	0.015	0.012	0.009	0.013	0.015	0.017	0.006	0.011	0.011	0.013	0.011	0.012
MLP (%)												
0	5.21	6.35	6.28^b^	5.46	2.48	2.17	2.80	2.74	2.72	4.18	3.48^b^	2.72
1	5.23	6.32	6.33^a^	5.46	2.51	2.16	2.81	2.76	2.70	4.17	3.52^a^	2.70
2	5.21	6.34	6.36^a^	5.45	2.49	2.15	2.82	2.75	2.72	4.19	3.54^a^	2.69
*p* Value	0.11	0.08	<.0001	0.31	0.07	0.22	0.05	0.07	0.07	0.27	0.04	0.10
SEM	0.015	0.012	0.009	0.013	0.015	0.017	0.006	0.011	0.011	0.013	0.011	0.012
Interaction effects												
no GTP + no MLP	5.32	6.31	6.17^c^	5.45	2.59	2.12	2.79	2.72	2.73	4.20	3.38^c^	2.73
1% GTP + no MLP	5.34	6.32	6.34^ab^	5.43	2.61	2.12	2.80	2.72	2.73	4.21	3.54^ab^	2.71
2% GTP + no MLP	5.31	6.35	6.33^b^	5.44	2.60	2.14	2.79	2.74	2.71	4.20	3.53^ab^	2.70
no GTP + 1% MLP	5.30	6.34	6.30^b^	5.45	2.60	2.13	2.81	2.73	2.70	4.21	3.49^b^	2.72
1% GTP + 1% MLP	5.33	6.33	6.36^ab^	5.45	2.62	2.11	2.82	2.73	2.70	4.23	3.55^ab^	2.72
2% GTP + 1% MLP	5.31	6.35	6.33^b^	5.46	2.60	2.13	2.81	2.75	2.72	4.22	3.51^b^	2.71
no GTP + 2% MLP	5.30	6.32	6.34^ab^	5.43	2.59	2.12	2.79	2.74	2.73	4.20	3.55^ab^	2.69
1% GTP + 2% MLP	5.33	6.33	6.32^b^	5.42	2.60	2.12	2.83	2.72	2.74	4.21	3.50^b^	2.70
2% GTP + 2% MLP	5.33	6.34	6.41^a^	5.43	2.61	2.14	2.83	2.73	2.74	4.21	3.58^a^	2.70
*p* Value	0.21	0.35	<.0001	0.32	0.19	0.28	0.26	0.30	0.34	0.52	<.0001	0.41
SEM	0.017	0.014	0.015	0.021	0.024	0.025	0.011	0.021	0.031	0.018	0.019	0.028

^a–c^Means within a column without a common superscript significantly differ (*p* < 0.05).

SEM: standard error of mean.

*0, 1, 2: different levels of GTP and MLP (%).

### 3.4 Intestinal morphology

The main and interaction effects of GTP and MLP on intestinal morphology are given in Table 6. About the main effects of GTP, the groups fed with 2% GTP had lower VH and VH: CD than the control or groups receiving 1% GTP (*p* < 0.05). Regarding the main effects of MLP, the groups fed with 2% MLP had lower VH than the groups receiving 1% MLP (*p* < 0.05). Concerning the interaction effects of GTP and MLP, the highest and lowest VH were observed in the treatments containing 1% GTP + 1% MLP, and 2% GTP + 2% MLP, respectively, which were significantly different from the control group (*p* < 0.05). Treatments: 1% GTP + 1% MLP, 1% GTP + no MLP and 2% GTP + no MLP showed the highest VH: CD, which were significantly higher than the control treatment (*p* < 0.05).

**TABLE 6:** Effects of different levels of green tea (GTP) and mulberry leaf powder (MLP) on jejunum morphology at 42 days of age.

Experimental treatments	Jejunum morphology characteristics				
Main effect^*^	Villus height (µm)	Villus width (µm)	Crypt depth (µm)	VH: CD	Muscle thickness (µm)
GTP (%)					
0	1171.36^a^	147.37	251.34	4.62^a^	262.14
1	1172.53^a^	143.92	247.17	4.65^a^	270.35
2	1167.71^b^	141.36	245.66	4.59^b^	265.41
*p* Value	0.03	0.08	0.06	0.01	0.16
SEM	0.940	1.41	2.44	0.009	3.24
MLP (%)					
0	1170.71^ab^	152.46	256.32	4.62	264.67
1	1172.73^a^	154.52	248.17	4.63	270.36
2	1169.13^b^	149.98	250.14	4.60	268.48
*p* Value	0.04	0.06	0.07	0.12	0.11
SEM	0.940	1.41	2.44	0.009	3.24
Interaction effects					
no GTP + no MLP	1169.94^bc^	148.74	255.67	4.56^d^	263.47
1% GTP + no MLP	1171.03^abc^	150.00	250.12	4.66^ab^	269.86
2% GTP + no MLP	1171.12^abc^	149.10	253.23	4.64^abc^	263.90
no GTP + 1% MLP	1172.61^ab^	151.02	252.76	4.62^abcd^	270.12
1% GTP + 1% MLP	1177.90^a^	152.56	249.90	4.69^a^	261.14
2% GTP + 1% MLP	1167.61^bc^	149.80	256.00	4.58^cd^	271.43
no GTP + 2% MLP	1171.42^abc^	148.47	252.10	4.62^abcd^	266.80
1% GTP + 2% MLP	1168.45^bc^	148.96	254.41	4.60^bcd^	265.76
2% GTP + 2% MLP	1164.22^c^	148.00	254.54	4.59^bcd^	270.78
*p* Value	0.01	0.06	0.08	<.0001	0.09
SEM	1.631	2.81	3.17	0.016	3.11

^a–d^Means within a column without a common superscript significantly differ (*p* < 0.05).

VH: CD: villus height to crypt depth ratio; SEM: standard error of mean.

^*^0, 1, 2: different levels of GTP and MLP (%).

## 4 DISCUSSION

Phytochemicals or bioactive ingredients in herbs, if used in sufficient amounts, will be beneficial for animal health (Adegbeye et al., 2020). Digestive enzymes and immune system function are stimulated by plant leaves (powdered or herbal extract), herbs, spices, and other plant‐based products (as a substitute for antibiotics) (Brenes & Roura, 2010). Former surveys have found that adding 10% and 20% fermented and unfermented mulberry leaves in the broilers’ diet considerably decreased the final body weight and digestibility of dry matter and crude protein (Has et al., 2013). One study showed that birds receiving different levels of MLP (0.1%, 0.2% and 0.3%) had better performance compared to the control group, especially at a concentration of 0.3% (Saenthaweesuk et al., 2010). In the current study, although there was no significant difference between the treatments in terms of DFI, the consumption of different levels of GTP and MLP increased the DWG, and these changes improved the FCR, so that the treatments receiving 2% GTP or MLP showed a better FCR compared to the control treatment during the grower and whole phases. The active compounds in herbals play a role in diminishing the population of harmful bacteria and increasing the population of beneficial bacteria in the digestive tract, and stimulating immunity. These beneficial bacteria have a significant role in improving the digestion of birds through the production of many essential enzymes in the digestive tract (Seidavi & Simoes, 2015). It is proposed that dietary supplementation of MLP boosts the broilers’ growth performance via improving intestinal tissue structure, digestive enzyme activities and nutrient digestibility (Ding et al., 2021). In the present study, GTP and MLP have stimulated humoral immunity and increased the VH: CD. As a result, performance improvement can be attributed to strengthening immunity, and enhancing digestion. A study showed that the green tea supplementation at 0.5%, 1% and 1.5% in the form of powder in the broilers’ diet has no effect on feed consumption (Hrncar & Bujko, 2017). Saraee et al. (2015) observed that the supplementation of fish oil (0.00%, 1.50% and 2.00%), GTP (0.00%, 1.00% and 1.50%) and their combination improved the feed intake, final body weight gain and FCR in broilers. Antioxidants from medicinal plants ameliorate carcass quality and performance by inhibiting reactive oxygen species or cell damage by free radicals that cause oxidative stress (Surai, 2019). The reasons for the difference in bird performance could be the source and composition of green tea (total, hydrolysable, and condensed polyphenols), the age and species of birds and the measured performance parameters.

Hrncar and Bujko (2017) showed that the various amounts of green tea did not affect carcass parameters, carcass products and most internal organs in broilers. In agreement with their study, we did not observe any significant difference between the treatments. Biswas and Wakita (2001) reported that the dressing percentage was not affected by the GTP added in four levels (0.5%, 0.75%, 1% and 1.5%). On the other hand, Olmo et al. (2012) found that the substitution of mulberry leaf meal at 10%, 20% and 30% decreases the weight of carcass, breast, thigh, leg and abdominal fat. Ding et al. (2021) reported that the supplementation of MLP and fermented MLP in the diet of broilers could decrease the abdominal fat percentage of broilers, which may be relevant to the regulation of active substances in mulberry leaves on the fat metabolism of broilers. The use of different parts of the plant, geographical location, stage of plant development, growth conditions and harvesting time can also cause changes in the effective substances, chemical composition and biological activity of plant agents. On the other hand, the amount of GTP and MLP used in the experiments and birds’ health conditions cause different responses (Burger et al., 1997).

One study checked the effect of green tea against coccidiosis in chickens and observed that there was increased cellular, and humoral immunity against coccidiosis, due to improvement in immunogenic response and immunoglobulin titres (total, IgG and IgM) (Abbas et al., 2017). The titre of antibodies in broilers reverberates the potency of humoral immunity. The superior the antibody titre, the more powerful the resistance of birds to infectious illness, and the less the chance of infectious diseases in birds (Gheorghiu et al., 1985). Immunoglobulins G, M and A are the primary immunoglobulins generated by activated B‐lymphocytes, indicating the humoral immune conditions. Amongst them, IgG is the principal member of antibodies in the serum and body fluids. It mostly takes part in the body's re‐humoral immune response (Lebacqverheyden et al., 1974). IgM is the first immunoglobulin is generated by activated B cells stimulated by antigen (Dekruyff et al., 1985). This type of immunoglobulin has a potent antigen‐binding capability and plays a substantial task in the early humoral immune defence (Gonzalez‐Suarez et al., 2009). In the present study, adding 2% GTP or MLP enhanced the total anti‐SRBC titre and IgG. Thereby, the highest total anti‐SRBC titre was obtained in treatments comprising 2% GTP + 2% MLP, 1% GTP + 1% MLP, 1% GTP + no MLP and no GTP + 2% MLP, which was considerably higher than the control and the diets containing 2% GTP + 2% MLP, 1% GTP + 1% MLP, 1% GTP + no MLP, no GTP + 2% MLP and 2% GTP + no MLP markedly increased the IgG titre. The experiment results of Song et al. (2016) showed that dietary supplementation of microencapsulated *Enterococcus faecalis* and the extract of *Camellia oleifera* seed increased IgG and IgA titres resulting in improving immune systems, reducing total cholesterol, and increasing serum biochemical parameters. Seidavi et al. (2017) reported that green tea had an advantageous impact on the immune response of broilers. This is because its polyphenols (mainly catechins), which have antibacterial and antioxidant properties, improve immune responses to influenza and Newcastle diseases (Santini & Novellino, 2017).

The biological foundation for growth and nutrient digestion and absorption in animals is the natural intestinal function and structure (El et al., 2015). Villus height, crypt depth and the ratio of villus height to crypt depth are important indexes to evaluate intestinal digestion and absorption in animals. The higher VH indicates the improvement of the intestinal health of birds, which in addition to a greater capacity to absorb nutrients, creates uniformity and integrity in the mucosa (Borsatti et al., 2020). Tohidifar et al. (2019) used different levels of MLP in broiler diets and they observed that the VH, VW and the CD of jejunum in treatments containing 1% of MLP were remarkably higher than the control group. In the current study, the highest VH and VH: CD were observed in broilers fed with the diets containing 1% GTP + 1% MLP, no% GTP + 1% MLP, 1% GTP + no MLP, 2% GTP + no MLP and no% GTP + 2% MLP. These results can be proof of the antioxidant impact of green tea on the intestinal tissue, and it is maybe the effect of green tea on apoptosis that leads to these differences in the morphology of the intestine. It has been shown that dietary antioxidants significantly protect the gut epithelial cells from stress, and improve the growth of epithelial cells (Miller et al., 2001). Previous studies revealed that as the VH increases, the intestinal digestive and absorptive functions elevate, therefore enhancing absorptive surface area, brush border enzymes’ expression, systems of nutrient transport, and body weight (Awad et al., 2009; Mohamed et al., 2014). Hasanpour et al. (2010) reported that the VH in the duodenum and jejunum compared to the control group significantly developed in the treatment containing green tea. Jelveh et al. (2022) used three levels of green tea extract (0.2, 0.3 and 0.4 g/kg) and powder (1, 2 and 3 g/kg) in the broilers’ diet and reported that green tea increased VH and VW in the different intestinal parts, compared with control. When studying the effect of orange essential oil on the histology of the intestine of broiler chickens, Erhan and Bolukbasi (2017) found that using 3 mL/kg of oil in the diets increased the VH. They related the increase in the VH to the antioxidant properties of the studied essential oil. It is known that green tea contains a high level of polyphenols, (Wu et al., 2016) and the main ingredient of polyphenols is catechins (Pasrija et al., 2015), which can efficaciously prevent the proliferation of pathogenic bacteria, and ameliorate the animal intestinal health (Hara, 2000).

## 5 CONCLUSIONS

The results showed that 2% green tea leaf powder or mulberry leaf powder in the broilers’ diet during the grower and whole phases caused a significant increase and decrease in daily weight gain and feed conversion ratio, respectively. Regarding the immune response, the use of 2% green tea leaf powder and mulberry leaf powder showed a higher titre for total anti‐sheep red blood cells and IgG. Meanwhile, the highest villus height to crypt depth ratio was observed in the groups fed with 1% green tea leaf powder + 1% mulberry leaf powder. In general, higher levels (2%) of green tea leaf powder and mulberry leaf powder are likely to improve the performance and immune response in broilers, while achieving the best villus height to crypt depth ratio, using a level of 1% green tea leaf powder without mulberry leaf powder seems to be sufficient.

## AUTHOR CONTRIBUTIONS

Fatemeh Aziz‐Aliabadi: conceptualisation; formal analysis; funding acquisition; investigation; methodology; project administration; resources; software; writing – original draft; writing – review & editing. Hadi Noruzi: conceptualisation; data curation; funding acquisition; investigation; methodology; project administration; resources; software; writing – original draft. Ahmad Hassanabadi: conceptualisation; investigation;

### ETHICS STATEMENT

All procedures were approved by the Animal Care and Use Committee of the Ferdowsi University of Mashhad, Mashhad, Iran.

### PEER REVIEW

The peer review history for this article is available at https://publons.com/publon/10.1002/vms3.1133.

## Data Availability

The data that support the findings of this study are available from the corresponding author upon reasonable request.

## Footnotes

1 General Linear Model.
